# Crystal structure and conformational analysis of doxorubicin nitrate

**DOI:** 10.1107/S2056989018002955

**Published:** 2018-02-28

**Authors:** Logesh Mathivathanan, Guang Yang, Fenfei Leng, Raphael G. Raptis

**Affiliations:** a Department of Chemistry and Biochemistry and Biomolecular Sciences Institute, Florida International University, 11200 SW 8th St, Miami, FL 33199, USA; b College of Chemistry and Molecular Engineering, Zhengzhou University, Zhengzhou, Henan, 450001, People’s Republic of China

**Keywords:** doxorubicin, anthracycline, conformation, inter­calation, crystal structure

## Abstract

Crystal structure determination of doxorubicin nitrate, (DoxH)NO_3_, systematic name (7*S*,9*S*)-7-{[(2*R*,4*S*,5*S*,6*S*)-4-azaniumyl-5-hy­droxy-6-methyl­oxan-2-yl]­oxy}-6,9,11-trihy­droxy-9-(2-hy­droxy­acet­yl)-4-meth­oxy-8,10-di­hydro-7*H*-tetra­cen-5,12-dione nitrate, shows two formula units present in the asymmetric unit. In the crystal lattice, hydrogen-bonded pairs of (DoxH^+^) cations and segregation of the aglycone and sugar moieties are observed. Inspection of mol­ecular overlays reveals that the conformation of (DoxH)NO_3_ resembles that of DNA-inter­calated, but not of protein-docked (DoxH)^+^. The structure was refined as a two-component twin.

## Abstract 2

The conformations of the two free doxorubicin (DoxH^+^) cations present in the crystal structure of the title compound and (Dox) bound to proteins and DNA are compared.

## Chemical context   

Since its discovery and isolation by genetic mutation of *Streptomyces peucetius* in 1969 (Arcamone *et al.*, 1969 ▸), the anthracycline anti­biotic doxorubicin [(Dox); trade name adriamycin] has become one of the most potent and widely used drugs in cancer chemotherapy (Denel-Bobrowska & Marczak, 2017 ▸; Cagel *et al.*, 2017 ▸; Cappetta *et al.*, 2018 ▸). Extensive studies of the anti­cancer activities of doxorubicin (Weiss, 1992 ▸; Shafei *et al.*, 2017 ▸) have led to FDA approval for the treatment of cancer forms, such as breast (Shafei *et al.*, 2017 ▸), ovarian (Duggan & Keating, 2011 ▸) and small-cell lung cancer (López-González *et al.*, 2013 ▸). The anti­cancer action of doxorubicin is a consequence of its inter­calation into base pairs of double-stranded DNA and subsequent inhibition of human DNA topoisomerase II (Arcamone, 1981 ▸; Liu, 1989 ▸; Chaires, 1998 ▸; Yang & Wang, 1999 ▸; Jung & Reszka, 2001 ▸). Although a few crystal structures of doxorubicin bound to DNA, enzymes and proteins have been reported, to the best of our knowledge, there is no crystal structure determination of doxorubicin itself in the literature. A Cambridge Structural Database (CSD version 5.38; Groom *et al.*, 2016 ▸) search for the doxorubicin skeleton structure gave only two hits for hydro­chloride salts of N- and O-substituted variants [CSD entries ADRMVL (Eckle & Stezowski, 1980 ▸) and BUJZIP (Eckle & Stezowski, 1983 ▸)]. Even for daunorubicin (also known as daunomycin), a closely related anthracycline anti­biotic, only the crystal structures of its hydro­chloride solvates have been reported (Neidle & Taylor, 1977 ▸; Courseille *et al.*, 1979 ▸). In the absence of a high-resolution crystal structure, researchers have so far relied on computational and solution studies to ascertain the preferred conformational geometry of (Dox) (Zhu *et al.*, 2010 ▸; Agrawal *et al.*, 2009 ▸; Barthwal *et al.*, 2008 ▸).
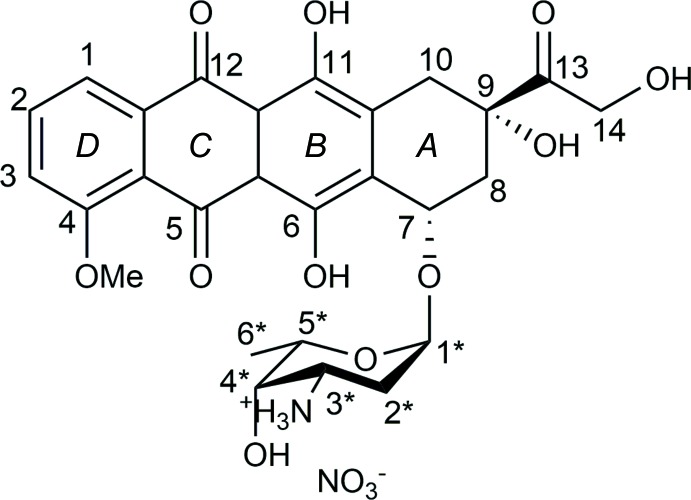


In order to probe and improve the activity of (Dox), several derivatives have been studied (Post *et al.*, 2005 ▸). Metal complexation to doxorubicin is known to alter its pharmaceutical activity and several Fe, Mn, Pt and Sn derivatives of the anthracycline have been studied with regard to their anti­cancer activities (Ming, 2003 ▸). (DoxH)^+^-functionalized iron oxide nanoparticles have been studied as cancer thera­nostics (Yu *et al.*, 2008 ▸). With a similar objective, we have attempted to coordinate Ag^+^ to (Dox). However, mixing stoichiometric amounts of (DoxH)Cl and AgNO_3_ in the presence of Et_3_N yielded only the nitrate derivative of (Dox) as (DoxH)NO_3_ in crystalline form. In this article, we report the 0.80 Å resolution crystal structure determination of doxorubicin nitrate and analyze and compare conformational details.

## Structural commentary   

**Figure 1:**
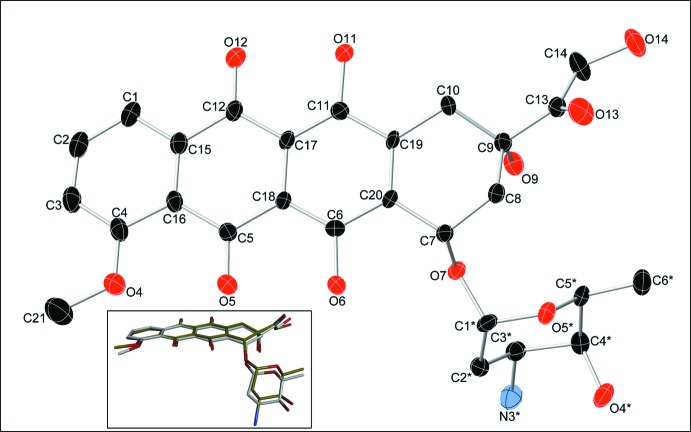
Mol­ecular structure of (DoxH)^+^ showing the atom-labeling scheme. Only one of the mol­ecules present in the asymmetric unit is shown, with displacement ellipsoids drawn at the 40% probability level. H atoms are not presented for clarity. Inset: Mol­ecular overlay of the two crystallographically independent (DoxH)^+^ moieties present in the asymmetric unit. The molecular overlay was performed using the function available within the Discovery Studio Visualizer Suite. The target chosen was one of the (Dox) units from the crystal structure, with the H atoms ignored.

The title compound crystallizes in the chiral monoclinic *P*2_1_ space group with two protonated doxorubicin cations (DoxH^+^) and two nitrate anions in the asymmetric unit. The (DoxH)^+^ cations consist of an aglycone, containing three approximately planar fused rings (the root-mean-square deviations of the six rings in the asymmetric unit are between 0.009 and 0.027 Å, *B*–*D*; the atom-numbering scheme and ring labels are shown in the scheme), and a sugar moiety in a chair conformation attached to ring *A*. Two nitrate ions hold pairs of cations with their fused rings at an approximately right angle to each other [86.4 (4)° between C1–C20 and N62(O64–O66)]. The two cations present in the asymmetric unit are rather similar, exhibiting insignificant differences (Fig. 1 ▸).

In 2010, Zhu and co-workers published a detailed conformational analysis of anthracycline anti­biotics, including doxorubicin, based on previously published (Dox)–protein and (Dox)–DNA complexes as well as DFT calculations (Zhu *et al.*, 2010 ▸). The analysis identified three important doxorubicin conformational domains: (1) the aromatic ring system, (2) the functional group at C9 and (3) at C7 relating to the aminal linkage:

(1) The aromatic anthracycline ring system does not vary significantly in any of the DNA-bound (Dox) structures and in the structure in this study. A somewhat more pronounced variation is encountered in protein-bound-(Dox), such as the one in 4dx7 or 4mra (*vide infra*). Based on the B3LYP level of theory, Zhu *et al.* have proposed four types of stable conformational isomers, with type I tautomer – forming two hydrogen bonds between C5—O and C6—OH and between C12—O and C11—OH – being the preferred one. The crystal structure in the present report confirms this prediction.

(2) The C8 carbon can either be above or below the anthracycline planes; in this structure, C8 is above the plane and the C19—C20—C7—C8 torsion angles are 16.6 (6)° and 17.5 (7)° (Table 1 ▸). This is in the expected range for an inter­calating (Dox), but significantly deviates from that found in a protein-bound (Dox). The conformation at C9 is similar to that at C8, as C9 is almost coplanar the anthracycline plane [C20—C19—C10—C9 torsion angles are 18.9 (6) and 19.2 (6)°]. More dramatic variations between the conformations of C8 and C9 are observed in the protein-bound (Dox) (5mra), where their torsion angles are 47.75 and −49.70°, respectively. According to a study based on resonant mol­ecular dynamic calculations and NMR experiments, the conformation with a C7—O7—C1*—C2* torsion angle of 142–143° was found to be biologically relevant (Barthwal *et al.*, 2008 ▸; Agrawal *et al.*, 2009 ▸). However, this seems to be only applicable to DNA-inter­calated (Dox). Protein-bound (Dox) have a wider range of torsion angles, for example, 88.43° in sorcin-bound (Dox), to 150.82° in AcrB-bound (Dox). The (Dox) structure in the present study has torsion angles of 161.6 (5) and 162.6 (4)°.

(3) The C7-connected daunosamine is the most flexible conformational entity in (Dox). The N3*—O7(C5) distance (2.74–8.50 Å) determines the conformational diversity. In the present structure, the corresponding distances are 7.947 (1) and 8.042 (2) Å, which are on the longer end of the spectrum. The presence of the nitrate ions between the two (Dox) fragments of the asymmetric unit influences this distance greatly.

## Supra­molecular features   

**Figure 2:**
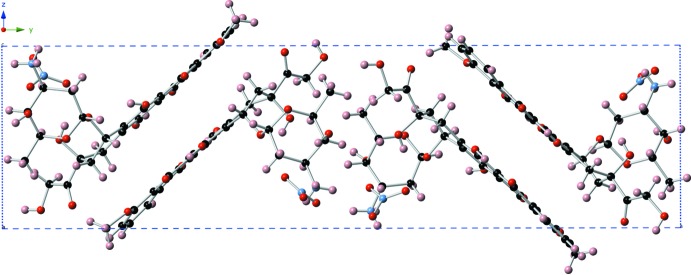
Mol­ecular packing diagram of (DoxH)NO_3_ viewed parallel to the crystallographic *a* axis.

The ammonium group forms hydrogen bonds with nitrate counter-ions with N⋯O distances of 2.836 (8), 2.876 (9) and 2.865 (8) Å (Table 2 ▸). The crystal structure is further stabilized by an extensive network of inter- and intra­molecular O—H⋯O and N—H⋯O hydrogen bonds (Table 2 ▸), in addition to two inter­molecular π–π inter­actions between the *C* and *D* rings of the aglycone moiety [centroid-to-centroid distances: 3.526 (3) and 3.694 (4) Å, Figs. 2 ▸ and 3 ▸] and a C—H⋯π inter­action with *Cg*2 [*Cg*2 is the centroid of the C1–C4/C15/C16 ring; C⋯*Cg* distance 3.556 (7) Å].

## Database survey   

Table 3 ▸ lists the published crystal structures of macromolecules with (Dox) as the ligand (DM2 ligand code in PDB; Berman *et al.*, 2000 ▸). To analyze the significance of the new (DoxH)NO_3_ crystal structure, structural comparisons were made by performing mol­ecular overlays (Dassault Systèmes BIOVIA, 2017 ▸) of the current structure with published (Dox)-bound protein/DNA structures, which resulted in the following observations: (1) The most important functional groups are, understandably, the amino group of the daunosamine moiety and the hydroxyl group of the glycolic site; (2) (Dox) binds in the DNA minor groove and (3) while the crystal structure reported here is by and large quite similar to the one of (Dox) bound in DNA, significant conformational differences are prominent in comparison to protein-bound (Dox), mainly because of differences in hydrogen-bond donors present in proteins.

**Figure 4:**
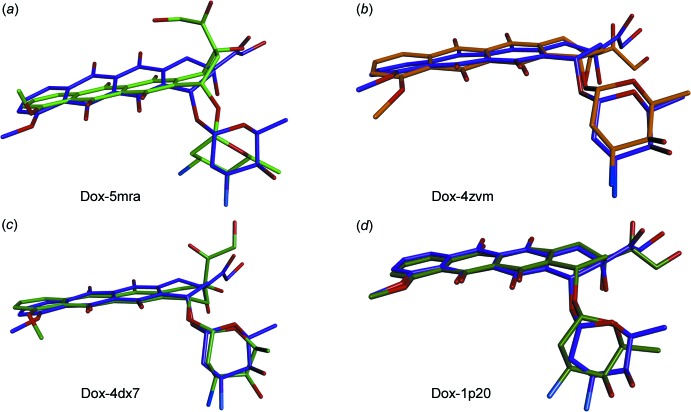
Representative mol­ecular overlays of doxorubicin from this study (purple) and the ones from the literature.

Binding of (Dox) to sorcin, a calcium-binding protein that causes multidrug resistance (MDR) in human tumors, impairs cell death. Sorcin is overexpressed in human tumors and MDR cancers. Two sites, designated as pocket 1 and pocket 2, were found to bind (Dox), which was modeled satisfactorily at pocket 1, but not at pocket 2. The mol­ecular overlay in Fig. 4 ▸ *a* shows the significant differences in the conformation: meth­oxy and the glycolic units are significantly rotated from their native state. On the contrary, DNA-bound (inter­calated) (Dox) and (Dox) in this study do not differ significantly in their conformations, as shown in (Dox)-1p20 in Fig. 4 ▸ below.

In another study, three mol­ecules of (Dox) were found to bind the AcrB protein (PDB accession 4dx7; Eicher *et al.*, 2012 ▸). Although the conformational differences are not as stark as they were in sorcin, the rotation now being about the bond between glycol-O carbon and the *A* ring [(Dox)-4dx7 in Fig. 5]. In the neurotoxin BoNT/B-(Dox) complex, O13 and O14 of the aglycone inter­act with the toxin and is stacked between Trp1261 and His1240 (Eswaramoorthy *et al.*, 2001 ▸). All the O and H atoms of the structure are hydrogen-bonded with various amino acid residues of the neurotoxin. The conformational changes in this complex are minimal, similar to DNA-bound (Dox).

## Synthesis and crystallization   

By mixing an ethano­lic solution of doxorubicin hydro­chloride (3 mg, 0.005 mmol), abbreviated as (DoxH)Cl, Et_3_N and an MeCN solution of AgNO_3_ (1.7 mg, 0.01 mmol), an orange solution was obtained. This was allowed to evaporate to near dryness to afford an orange powder. The orange powder was then dissolved in EtOH. After filtration, the filtrate was layered with Et_2_O. Red sheet-like crystals of (DoxH)NO_3_ were obtained in two weeks.

## Refinement   

Crystal data, data collection and structure refinement details are summarized in Table 4 ▸. All H atoms were positioned geometrically and refined using a riding model: O—H = 0.82, N—H = 0.89 and C—H = 0.93–0.98 Å with *U*_iso_(H) = 1.2 or 1.5*U*_eq_(parent atom). The structure was refined as a two-component twin (matrix to transform one domain into the other: (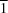 0 0 0 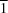 0 1 0 1); BASF = 0.3072). Atoms marked with a star correspond to the pyran­ose ring, following a numbering convention previously described for (Dox) (Eswaramoorthy *et al.*, 2001 ▸).

## Supplementary Material

Crystal structure: contains datablock(s) I. DOI: 10.1107/S2056989018002955/wm5435sup1.cif

Structure factors: contains datablock(s) I. DOI: 10.1107/S2056989018002955/wm5435Isup2.hkl

Supporting information file. DOI: 10.1107/S2056989018002955/wm5435Isup3.cdx

CCDC reference: 1815074

Additional supporting information:  crystallographic information; 3D view; checkCIF report

## Figures and Tables

**Figure 3:**
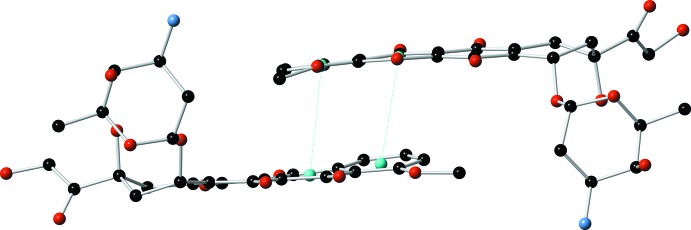
Representation highlighting the π–π inter­actions between *C* and *D* rings of the aglycone moieties. Turquoise spheres indicate centroids.

**Table 1:** Conformational parameters (°) of one of the (DoxH)^+^ cations present in the title compound, (DoxH)NO_3_, and representative examples from the literature

	(DoxH)NO_3_	AcrB-(Dox) (4dx7)^*a*^	Sorcin-(Dox) (5mra)^*b*^	DNA-(Dox) (1p20)^*c*^
C7—O7—C1*—C2*	161.6 (5), 162.6 (4)	150.82	88.43	144.36
C8 conformation C19—C20—C7—C8	16.6 (6), 17.5 (7)	−18.93	47.75	8.92
C9 conformation C20—C19—C10—C9	18.9 (6), 19.2 (6)	−9.27	−49.70	22.91
O7—N3*	7.947 (1), 8.042 (1)	7.636	6.433	6.481

Notes: (*a*) Eicher *et al.* (2012 ▸); *b* Genovese *et al.* (2017 ▸); *c* Howerton *et al.* (2003 ▸).

**Table 2:** Hydrogen-bond geometry (Å, °) *Cg*2 is the centroid of the C1–C4/C15/C16 ring.

*D*—H⋯*A*	*D*—H	H⋯*A*	*D*⋯*A*	*D*—H⋯*A*
O6—H6⋯O5	0.82	1.81	2.526 (6)	146
O11—H11⋯O12	0.82	1.80	2.526 (6)	146
O14—H14⋯O65^i^	0.82	2.07	2.779 (9)	145
N3*—H3*A⋯O13^ii^	0.89	2.00	2.874 (7)	167
N3*—H3*C⋯O63	0.89	1.99	2.865 (8)	168
O41—H41⋯O42	0.82	1.82	2.537 (6)	146
O44—H44⋯O14^iii^	0.82	2.08	2.888 (6)	168
O55—H55⋯O4*	0.82	1.93	2.724 (6)	163
N54—H54*A*⋯O43^iv^	0.89	2.18	2.890 (7)	136
N54—H54*A*⋯O44^iv^	0.89	2.05	2.843 (7)	147
N54—H54*B*⋯O62	0.89	1.99	2.836 (8)	159
N54—H54*C*⋯O64	0.89	2.05	2.876 (9)	155
C38—H38*B*⋯*Cg*2^v^	0.97	2.64	3.556 (7)	157

Symmetry codes: (i) 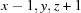; (ii) 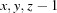; (iii) 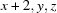; (iv) 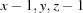; (v) 
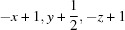
.

**Table 3:** Selected RCSB-PDB entries with (Dox) (*DM2*) as the ligand

PDB accession (Reference)	Macromolecule(*s*)	Resolution (Å)
5MRA (Genovese *et al.*, 2017 ▸)	Sorcin (protein)	3.74
4ZVM (Leung & Shilton, 2015 ▸)	Ribsyldi­hydro­nicotinamide de­hydrogenase	1.97
4DX7 (Eicher *et al.*, 2012 ▸)	Acriflavine resistance protein B (protein) DARPIN (protein)	2.25
2DR6 (Murakami *et al.*, 2006 ▸)	AcrB (protein)	3.3
1P20 (Howerton *et al.*, 2003 ▸)	DNA	1.34
1I1E (Eswaramoorthy *et al.*, 2001 ▸)	Botulinium Neurotoxin Type B (protein)	2.5
151D (Lipscomb *et al.*, 1994 ▸)	DNA	1.4
1DA9 (Leonard *et al.*, 1993 ▸)	DNA	1.7
1D12 (Frederick *et al.*, 1990 ▸)	DNA	1.7

**Table 4:** Experimental details

Crystal data	
Chemical formula	C_27_H_30_NO_11_·NO_3_
*M*_r_	606.53
Crystal system, space group	Monoclinic, *P*2_1_
Temperature (K)	298
*a*, *b*, *c* (Å)	8.3169 (12), 34.280 (5), 10.1010 (14)
β (°)	114.293 (4)
*V* (Å^3^)	2624.8 (6)
*Z*	4
Radiation type	Mo *K*α
μ (mm^−1^)	0.13
Crystal size (mm)	0.24 × 0.10 × 0.05

Data collection	
Diffractometer	Bruker D8 Quest CMOS
Absorption correction	Multi-scan (*SADABS*; Bruker, 2016 ▸)
*T*_min_, *T*_max_	0.685, 0.745
No. of measured, independent and observed [*I* > 2σ(*I*)] reflections	33597, 10764, 7299
*R*_int_	0.049
(sin θ/λ)_max_ (Å^−1^)	0.626

Refinement	
*R*[*F* ^2^ > 2σ(*F* ^2^)], *wR*(*F* ^2^), *S*	0.050, 0.093, 1.01
No. of reflections	10764
No. of parameters	792
No. of restraints	1
H-atom treatment	H-atom parameters constrained
Δρ_max_, Δρ_min_ (e Å^−3^)	0.24, −0.18
Absolute structure	Flack *x* determined using 2632 quotients [(*I* ^+^)−(*I* ^−^)]/[(*I* ^+^)+(*I* ^−^)] (Parsons *et al.*, 2013 ▸).
Absolute structure parameter	−0.3 (4)

Computer programs: *APEX3* and *SAINT* (Bruker, 2016 ▸), *SHELXT* (Sheldrick, 2015*b* ▸), *SHELXL* (Sheldrick, 2015*a* ▸), *OLEX2* (Dolomanov *et al.*, 2009 ▸) and *CrystalMaker* (Palmer, 2017 ▸).

## supplementary crystallographic information

### Crystal data

**Table:**

C_27_H_30_NO_11_^+^·NO_3_^−^	*F*(000) = 1272
*M**_r_* = 606.53	*D*_x_ = 1.535 Mg m^−^^3^
Monoclinic, *P*2_1_	Mo *K*α radiation, λ = 0.71073 Å
*a* = 8.3169 (12) Å	Cell parameters from 9978 reflections
*b* = 34.280 (5) Å	θ = 2.9–26.4°
*c* = 10.1010 (14) Å	µ = 0.13 mm^−^^1^
β = 114.293 (4)°	*T* = 298 K
*V* = 2624.8 (6) Å^3^	Sheet, red
*Z* = 4	0.24 × 0.10 × 0.05 mm

### Data collection

**Table:**

Bruker D8 Quest CMOS diffractometer	7299 reflections with *I* > 2σ(*I*)
Detector resolution: 10.42 pixels mm^-1^	*R*_int_ = 0.049
φ and ω scans	θ_max_ = 26.4°, θ_min_ = 2.8°
Absorption correction: multi-scan (*SADABS*; Bruker, 2016)	*h* = −10→10
*T*_min_ = 0.685, *T*_max_ = 0.745	*k* = −42→42
33597 measured reflections	*l* = −12→12
10764 independent reflections	

### Refinement

**Table:**

Refinement on *F*^2^	Hydrogen site location: inferred from neighbouring sites
Least-squares matrix: full	H-atom parameters constrained
*R*[*F*^2^ > 2σ(*F*^2^)] = 0.050	*w* = 1/[σ^2^(*F*_o_^2^) + (0.0405*P*)^2^] where *P* = (*F*_o_^2^ + 2*F*_c_^2^)/3
*wR*(*F*^2^) = 0.093	(Δ/σ)_max_ = 0.001
*S* = 1.01	Δρ_max_ = 0.24 e Å^−^^3^
10764 reflections	Δρ_min_ = −0.18 e Å^−^^3^
792 parameters	Absolute structure: Flack *x* determined using 2632 quotients [(*I*^+^)-(*I*^-^)]/[(*I*^+^)+(*I*^-^)] (Parsons *et al.*, 2013).
1 restraint	Absolute structure parameter: −0.3 (4)
Primary atom site location: dual	

### Special details

**Table:**

Geometry. All esds (except the esd in the dihedral angle between two l.s. planes) are estimated using the full covariance matrix. The cell esds are taken into account individually in the estimation of esds in distances, angles and torsion angles; correlations between esds in cell parameters are only used when they are defined by crystal symmetry. An approximate (isotropic) treatment of cell esds is used for estimating esds involving l.s. planes.
Refinement. Refined as a 2-component twin.

### Fractional atomic coordinates and isotropic or equivalent isotropic displacement parameters (Å^2^)

**Table:**

	*x*	*y*	*z*	*U*_iso_*/*U*_eq_	
O4	−0.0502 (7)	0.18514 (13)	0.0931 (5)	0.0585 (13)	
O4*	0.6155 (6)	0.48108 (13)	0.5178 (5)	0.0481 (11)	
H4*	0.645686	0.465763	0.469977	0.072*	
O5	0.1037 (6)	0.24134 (12)	0.2718 (5)	0.0513 (12)	
O5*	0.5027 (5)	0.42141 (11)	0.6516 (4)	0.0373 (10)	
O6	0.2581 (5)	0.29480 (12)	0.4514 (4)	0.0385 (10)	
H6	0.249029	0.277163	0.393904	0.058*	
O7	0.2617 (5)	0.38118 (10)	0.5142 (4)	0.0305 (9)	
O9	0.0248 (6)	0.42485 (12)	0.6025 (4)	0.0404 (10)	
H9	0.008204	0.413375	0.526779	0.061*	
O11	−0.3440 (5)	0.31738 (12)	0.5332 (5)	0.0456 (12)	
H11	−0.425673	0.303697	0.479458	0.068*	
O12	−0.4933 (6)	0.26575 (13)	0.3438 (5)	0.0518 (12)	
O13	0.1964 (6)	0.41696 (14)	0.9652 (5)	0.0592 (13)	
O14	−0.0341 (8)	0.47530 (13)	0.9372 (5)	0.0622 (13)	
H14	−0.003156	0.463169	1.013720	0.093*	
N3*	0.3633 (7)	0.46242 (16)	0.2253 (6)	0.0500 (14)	
H3*A	0.297507	0.447837	0.149414	0.060*	
H3*B	0.475149	0.461670	0.236527	0.060*	
H3*C	0.324774	0.486934	0.210406	0.060*	
C1	−0.5146 (9)	0.21495 (18)	0.1278 (7)	0.0444 (17)	
H1	−0.618929	0.221160	0.136119	0.053*	
C1*	0.4370 (7)	0.39312 (17)	0.5417 (7)	0.0370 (15)	
H1*	0.513936	0.370188	0.572263	0.044*	
C2	−0.5114 (10)	0.18808 (19)	0.0281 (7)	0.0497 (18)	
H2	−0.617255	0.176785	−0.034084	0.060*	
C2*	0.4371 (9)	0.40795 (16)	0.4007 (7)	0.0420 (16)	
H2*A	0.557785	0.409799	0.410350	0.050*	
H2*B	0.375553	0.389406	0.323876	0.050*	
C3	−0.3624 (10)	0.17727 (19)	0.0160 (7)	0.0468 (18)	
H3	−0.367212	0.158116	−0.050736	0.056*	
C3*	0.3507 (8)	0.44697 (18)	0.3592 (6)	0.0374 (15)	
H3*	0.225666	0.444100	0.339457	0.045*	
C4	−0.2009 (9)	0.19443 (17)	0.1022 (7)	0.0403 (16)	
C4*	0.4339 (8)	0.47586 (18)	0.4843 (6)	0.0384 (15)	
H4*A	0.372292	0.500959	0.459076	0.046*	
C5	−0.0353 (8)	0.24605 (16)	0.2879 (6)	0.0321 (14)	
C5*	0.4159 (8)	0.45854 (16)	0.6152 (6)	0.0353 (14)	
H5*	0.290226	0.454630	0.591093	0.042*	
C6	0.1027 (7)	0.29952 (16)	0.4623 (6)	0.0286 (13)	
C6*	0.4922 (10)	0.48356 (18)	0.7488 (7)	0.0515 (18)	
H6*A	0.464805	0.472349	0.824116	0.077*	
H6*B	0.442708	0.509284	0.726627	0.077*	
H6*C	0.617912	0.485033	0.780942	0.077*	
C7	0.2549 (7)	0.35565 (15)	0.6252 (6)	0.0283 (13)	
H7	0.360579	0.339180	0.661049	0.034*	
C8	0.2488 (7)	0.37850 (16)	0.7521 (6)	0.0291 (13)	
H8A	0.278066	0.361105	0.834527	0.035*	
H8B	0.337843	0.398804	0.779461	0.035*	
C9	0.0706 (7)	0.39708 (16)	0.7192 (6)	0.0307 (14)	
C10	−0.0675 (8)	0.36543 (16)	0.6807 (7)	0.0358 (15)	
H10A	−0.056823	0.352417	0.769223	0.043*	
H10B	−0.183339	0.377406	0.637884	0.043*	
C11	−0.2010 (8)	0.31119 (17)	0.5054 (6)	0.0315 (14)	
C12	−0.3558 (8)	0.26088 (16)	0.3247 (6)	0.0343 (14)	
C13	0.0784 (8)	0.42099 (16)	0.8475 (7)	0.0328 (14)	
C14	−0.0640 (10)	0.4493 (2)	0.8234 (7)	0.061 (2)	
H14A	−0.082796	0.464209	0.736602	0.074*	
H14B	−0.171787	0.435043	0.805093	0.074*	
C15	−0.3541 (8)	0.23265 (16)	0.2165 (7)	0.0335 (14)	
C16	−0.1969 (8)	0.22379 (16)	0.2027 (6)	0.0325 (14)	
C17	−0.1987 (7)	0.28312 (16)	0.4090 (6)	0.0290 (13)	
C18	−0.0431 (7)	0.27627 (15)	0.3858 (6)	0.0277 (12)	
C19	−0.0547 (7)	0.33577 (15)	0.5783 (6)	0.0288 (13)	
C20	0.0949 (7)	0.32995 (16)	0.5542 (6)	0.0264 (12)	
C21	−0.0478 (11)	0.1538 (2)	0.0032 (9)	0.076 (3)	
H21A	−0.133917	0.158353	−0.093937	0.115*	
H21B	−0.075012	0.130013	0.039518	0.115*	
H21C	0.067198	0.151890	0.002926	0.115*	
O34	0.9753 (7)	0.82204 (16)	−0.0230 (6)	0.0754 (16)	
O35	1.0122 (6)	0.76876 (14)	0.1649 (5)	0.0599 (14)	
O36	1.0308 (5)	0.71127 (12)	0.3215 (5)	0.0456 (12)	
H36	0.989301	0.689262	0.313851	0.068*	
O37	1.1071 (5)	0.62664 (11)	0.3901 (4)	0.0343 (10)	
O39	1.4642 (7)	0.58445 (12)	0.5127 (5)	0.0506 (12)	
H39	1.386575	0.591626	0.435418	0.076*	
O41	1.7277 (5)	0.69683 (12)	0.4310 (5)	0.0428 (11)	
H41	1.755740	0.711119	0.378857	0.064*	
O42	1.6881 (6)	0.75101 (12)	0.2491 (5)	0.0470 (12)	
O43	1.6156 (7)	0.60300 (13)	0.8710 (5)	0.0591 (14)	
O44	1.8716 (6)	0.55690 (13)	0.8977 (4)	0.0508 (12)	
H44	1.895492	0.533742	0.896090	0.076*	
O55	0.7047 (6)	0.53757 (13)	0.3706 (5)	0.0498 (11)	
H55	0.698298	0.518809	0.418573	0.075*	
O56	0.9570 (5)	0.59384 (11)	0.5073 (4)	0.0373 (10)	
N54	0.7120 (7)	0.54394 (16)	0.0945 (5)	0.0469 (14)	
H54A	0.721658	0.553201	0.015785	0.056*	
H54B	0.607796	0.550745	0.092677	0.056*	
H54C	0.720612	0.518053	0.095890	0.056*	
C31	1.4914 (9)	0.80230 (17)	0.0381 (7)	0.0429 (16)	
H31	1.607255	0.797568	0.051785	0.052*	
C32	1.3907 (11)	0.8297 (2)	−0.0621 (7)	0.0523 (19)	
H32	1.440145	0.843813	−0.114745	0.063*	
C33	1.2187 (11)	0.83629 (19)	−0.0847 (7)	0.0527 (19)	
H33	1.151700	0.854418	−0.153945	0.063*	
C34	1.1447 (9)	0.81639 (18)	−0.0061 (7)	0.0453 (17)	
C35	1.1642 (9)	0.76388 (18)	0.1767 (7)	0.0394 (15)	
C36	1.2008 (8)	0.70888 (16)	0.3421 (6)	0.0324 (14)	
C37	1.2158 (8)	0.65236 (16)	0.5045 (6)	0.0329 (14)	
H37	1.138142	0.667903	0.535057	0.040*	
C38	1.3426 (8)	0.63079 (18)	0.6346 (6)	0.0361 (14)	
H38A	1.281283	0.609083	0.654825	0.043*	
H38B	1.381380	0.648111	0.717901	0.043*	
C39	1.5042 (8)	0.61522 (16)	0.6169 (6)	0.0341 (14)	
C40	1.5997 (8)	0.64825 (18)	0.5799 (7)	0.0387 (15)	
H40A	1.668966	0.662410	0.668291	0.046*	
H40B	1.680911	0.637217	0.543365	0.046*	
C41	1.5543 (8)	0.70137 (16)	0.3987 (6)	0.0312 (14)	
C42	1.5295 (9)	0.75381 (16)	0.2251 (7)	0.0344 (15)	
C43	1.6279 (8)	0.59617 (17)	0.7599 (7)	0.0345 (14)	
C44	1.7663 (9)	0.56963 (18)	0.7555 (7)	0.0424 (16)	
H44A	1.712099	0.547390	0.693898	0.051*	
H44B	1.838642	0.583201	0.715593	0.051*	
C45	1.4174 (8)	0.78187 (17)	0.1183 (6)	0.0345 (14)	
C46	1.2408 (9)	0.78777 (18)	0.0958 (6)	0.0381 (15)	
C47	1.4527 (8)	0.72972 (16)	0.3014 (6)	0.0316 (14)	
C48	1.2731 (8)	0.73373 (15)	0.2749 (6)	0.0291 (13)	
C49	1.4795 (8)	0.67671 (15)	0.4689 (6)	0.0301 (14)	
C50	1.3049 (8)	0.67947 (15)	0.4398 (6)	0.0308 (14)	
C51	0.8621 (12)	0.8447 (3)	−0.1417 (9)	0.085 (3)	
H51A	0.862363	0.834571	−0.230158	0.128*	
H51B	0.902539	0.871270	−0.128992	0.128*	
H51C	0.744427	0.843774	−0.146502	0.128*	
C52	0.9421 (8)	0.61882 (18)	0.3907 (7)	0.0393 (15)	
H52	0.892273	0.643636	0.404134	0.047*	
C53	0.8221 (8)	0.60273 (18)	0.2455 (7)	0.0451 (17)	
H53A	0.700737	0.605672	0.233108	0.054*	
H53B	0.836898	0.617729	0.169811	0.054*	
C54	0.8571 (8)	0.56056 (18)	0.2284 (6)	0.0384 (15)	
H54	0.968632	0.558537	0.217536	0.046*	
C55	0.8725 (8)	0.53706 (18)	0.3605 (6)	0.0367 (15)	
H55A	0.905157	0.510103	0.350454	0.044*	
C56	1.0138 (8)	0.55541 (16)	0.4903 (6)	0.0341 (14)	
H56	1.121379	0.557499	0.473420	0.041*	
C57	1.0544 (9)	0.5327 (2)	0.6275 (7)	0.0515 (18)	
H57A	1.139700	0.546715	0.708267	0.077*	
H57B	1.101245	0.507665	0.619801	0.077*	
H57C	0.948384	0.529354	0.642242	0.077*	
O61	0.3752 (8)	0.59449 (18)	0.2043 (7)	0.0812 (17)	
O62	0.3580 (7)	0.5458 (2)	0.0653 (6)	0.092 (2)	
O63	0.2521 (7)	0.54165 (16)	0.2223 (6)	0.0678 (14)	
N61	0.3272 (8)	0.5614 (2)	0.1620 (6)	0.0525 (15)	
O64	0.6646 (9)	0.4626 (2)	0.1392 (8)	0.102 (2)	
O65	0.9171 (9)	0.4433 (3)	0.1708 (8)	0.138 (3)	
O66	0.7773 (11)	0.4163 (2)	0.2802 (8)	0.119 (3)	
N62	0.7890 (10)	0.4409 (2)	0.1966 (7)	0.0611 (17)	

### Atomic displacement parameters (Å^2^)

**Table:**

	*U*^11^	*U*^22^	*U*^33^	*U*^12^	*U*^13^	*U*^23^
O4	0.058 (3)	0.054 (3)	0.070 (3)	−0.007 (2)	0.032 (3)	−0.031 (3)
O4*	0.046 (3)	0.046 (3)	0.049 (3)	−0.020 (2)	0.016 (2)	−0.005 (2)
O5	0.046 (3)	0.051 (3)	0.068 (3)	−0.007 (2)	0.034 (3)	−0.022 (2)
O5*	0.038 (2)	0.032 (2)	0.035 (2)	−0.010 (2)	0.0085 (19)	−0.005 (2)
O6	0.034 (3)	0.039 (3)	0.046 (3)	−0.003 (2)	0.020 (2)	−0.013 (2)
O7	0.029 (2)	0.0262 (19)	0.037 (2)	−0.0035 (17)	0.0146 (19)	0.0007 (18)
O9	0.050 (3)	0.036 (2)	0.033 (2)	0.004 (2)	0.014 (2)	−0.001 (2)
O11	0.034 (3)	0.044 (3)	0.068 (3)	−0.010 (2)	0.030 (3)	−0.019 (2)
O12	0.035 (3)	0.053 (3)	0.072 (3)	−0.012 (2)	0.027 (2)	−0.021 (3)
O13	0.061 (3)	0.070 (3)	0.033 (3)	0.010 (3)	0.005 (3)	−0.014 (2)
O14	0.083 (4)	0.049 (3)	0.061 (3)	0.000 (3)	0.036 (3)	−0.022 (3)
N3*	0.050 (3)	0.054 (3)	0.041 (3)	−0.010 (3)	0.013 (3)	−0.001 (3)
C1	0.045 (4)	0.038 (4)	0.040 (4)	−0.013 (3)	0.008 (3)	−0.001 (3)
C1*	0.027 (3)	0.032 (3)	0.056 (4)	−0.003 (3)	0.021 (3)	0.002 (3)
C2	0.054 (5)	0.040 (4)	0.045 (4)	−0.013 (4)	0.011 (4)	−0.007 (3)
C2*	0.053 (4)	0.030 (3)	0.055 (4)	−0.009 (3)	0.035 (4)	−0.008 (3)
C3	0.064 (5)	0.042 (4)	0.032 (4)	−0.010 (4)	0.016 (4)	−0.009 (3)
C3*	0.035 (4)	0.046 (4)	0.031 (3)	−0.008 (3)	0.013 (3)	−0.004 (3)
C4	0.059 (5)	0.026 (3)	0.037 (4)	−0.001 (3)	0.022 (4)	−0.002 (3)
C4*	0.037 (4)	0.036 (3)	0.036 (4)	−0.001 (3)	0.009 (3)	−0.005 (3)
C5	0.037 (4)	0.029 (3)	0.033 (3)	−0.002 (3)	0.018 (3)	−0.001 (3)
C5*	0.036 (4)	0.028 (3)	0.041 (4)	−0.011 (3)	0.015 (3)	−0.007 (3)
C6	0.027 (3)	0.031 (3)	0.029 (3)	0.000 (3)	0.013 (3)	0.004 (3)
C6*	0.065 (5)	0.039 (4)	0.053 (4)	−0.015 (4)	0.026 (4)	−0.013 (3)
C7	0.030 (3)	0.021 (3)	0.036 (3)	−0.001 (3)	0.016 (3)	0.001 (3)
C8	0.026 (3)	0.029 (3)	0.027 (3)	−0.002 (3)	0.005 (3)	−0.001 (3)
C9	0.033 (4)	0.030 (3)	0.028 (3)	−0.001 (3)	0.011 (3)	−0.002 (3)
C10	0.037 (4)	0.030 (3)	0.043 (4)	−0.007 (3)	0.020 (3)	−0.014 (3)
C11	0.030 (4)	0.029 (3)	0.039 (3)	0.002 (3)	0.017 (3)	−0.001 (3)
C12	0.041 (4)	0.024 (3)	0.040 (4)	−0.007 (3)	0.018 (3)	−0.001 (3)
C13	0.039 (4)	0.023 (3)	0.038 (4)	−0.001 (3)	0.017 (3)	0.000 (3)
C14	0.067 (5)	0.066 (5)	0.050 (4)	0.006 (4)	0.022 (4)	−0.024 (4)
C15	0.041 (4)	0.023 (3)	0.034 (3)	−0.003 (3)	0.013 (3)	0.002 (3)
C16	0.047 (4)	0.020 (3)	0.032 (3)	−0.002 (3)	0.017 (3)	0.000 (3)
C17	0.029 (3)	0.022 (3)	0.035 (3)	−0.006 (3)	0.012 (3)	−0.002 (3)
C18	0.029 (3)	0.022 (3)	0.032 (3)	−0.004 (3)	0.013 (3)	−0.002 (3)
C19	0.030 (3)	0.017 (3)	0.039 (3)	−0.007 (3)	0.014 (3)	−0.005 (3)
C20	0.025 (3)	0.022 (3)	0.030 (3)	−0.006 (2)	0.009 (3)	−0.001 (3)
C21	0.077 (6)	0.079 (6)	0.070 (5)	0.009 (5)	0.025 (5)	−0.035 (5)
O34	0.073 (4)	0.090 (4)	0.065 (3)	0.034 (3)	0.030 (3)	0.042 (3)
O35	0.044 (3)	0.065 (3)	0.072 (3)	0.020 (3)	0.025 (3)	0.034 (3)
O36	0.032 (3)	0.041 (3)	0.068 (3)	0.002 (2)	0.024 (2)	0.012 (3)
O37	0.033 (2)	0.029 (2)	0.042 (2)	−0.0055 (19)	0.017 (2)	0.002 (2)
O39	0.067 (3)	0.041 (2)	0.035 (2)	0.015 (2)	0.012 (2)	−0.002 (2)
O41	0.033 (3)	0.045 (3)	0.055 (3)	0.006 (2)	0.023 (2)	0.016 (2)
O42	0.040 (3)	0.053 (3)	0.051 (3)	−0.006 (2)	0.021 (2)	0.009 (2)
O43	0.081 (4)	0.063 (3)	0.037 (3)	0.029 (3)	0.028 (3)	0.010 (2)
O44	0.057 (3)	0.051 (3)	0.040 (3)	0.012 (2)	0.017 (2)	0.012 (2)
O55	0.043 (3)	0.057 (3)	0.051 (3)	−0.011 (2)	0.022 (2)	0.019 (2)
O56	0.039 (3)	0.034 (2)	0.044 (2)	−0.001 (2)	0.022 (2)	0.005 (2)
N54	0.047 (3)	0.053 (3)	0.039 (3)	−0.006 (3)	0.016 (3)	0.005 (3)
C31	0.056 (4)	0.030 (3)	0.045 (4)	−0.008 (3)	0.024 (4)	0.003 (3)
C32	0.068 (5)	0.046 (4)	0.042 (4)	−0.014 (4)	0.022 (4)	0.007 (4)
C33	0.069 (5)	0.037 (4)	0.042 (4)	0.002 (4)	0.012 (4)	0.012 (3)
C34	0.053 (5)	0.035 (4)	0.049 (4)	0.010 (3)	0.022 (4)	0.005 (3)
C35	0.040 (4)	0.034 (4)	0.043 (4)	0.003 (3)	0.016 (3)	0.002 (3)
C36	0.026 (3)	0.029 (3)	0.043 (4)	−0.001 (3)	0.016 (3)	−0.006 (3)
C37	0.039 (4)	0.028 (3)	0.035 (3)	0.004 (3)	0.019 (3)	0.002 (3)
C38	0.041 (4)	0.041 (4)	0.033 (3)	0.001 (3)	0.023 (3)	0.006 (3)
C39	0.044 (4)	0.028 (3)	0.028 (3)	0.005 (3)	0.013 (3)	0.003 (3)
C40	0.035 (4)	0.047 (4)	0.038 (3)	0.010 (3)	0.018 (3)	0.009 (3)
C41	0.034 (4)	0.024 (3)	0.038 (3)	0.000 (3)	0.017 (3)	0.000 (3)
C42	0.042 (4)	0.028 (3)	0.037 (4)	−0.008 (3)	0.019 (3)	−0.009 (3)
C43	0.044 (4)	0.028 (3)	0.032 (3)	0.002 (3)	0.016 (3)	0.002 (3)
C44	0.054 (4)	0.036 (3)	0.035 (4)	0.007 (3)	0.016 (3)	0.001 (3)
C45	0.040 (4)	0.032 (3)	0.031 (3)	−0.003 (3)	0.014 (3)	0.002 (3)
C46	0.048 (4)	0.035 (4)	0.030 (3)	0.002 (3)	0.015 (3)	0.001 (3)
C47	0.038 (4)	0.024 (3)	0.035 (3)	0.000 (3)	0.016 (3)	0.001 (3)
C48	0.035 (4)	0.018 (3)	0.033 (3)	0.001 (3)	0.013 (3)	0.002 (3)
C49	0.041 (4)	0.019 (3)	0.034 (3)	0.004 (3)	0.019 (3)	0.002 (3)
C50	0.038 (4)	0.019 (3)	0.038 (3)	0.003 (3)	0.017 (3)	−0.002 (3)
C51	0.081 (6)	0.109 (7)	0.056 (5)	0.041 (5)	0.019 (5)	0.014 (5)
C52	0.030 (4)	0.036 (3)	0.054 (4)	0.005 (3)	0.020 (3)	0.015 (3)
C53	0.037 (4)	0.042 (4)	0.047 (4)	−0.003 (3)	0.008 (3)	0.019 (3)
C54	0.036 (4)	0.044 (4)	0.037 (3)	−0.001 (3)	0.016 (3)	0.008 (3)
C55	0.036 (4)	0.034 (3)	0.046 (4)	0.002 (3)	0.022 (3)	0.004 (3)
C56	0.032 (3)	0.035 (3)	0.037 (4)	0.002 (3)	0.016 (3)	0.013 (3)
C57	0.055 (4)	0.051 (4)	0.054 (4)	0.011 (4)	0.028 (4)	0.009 (4)
O61	0.093 (5)	0.055 (4)	0.092 (4)	0.003 (3)	0.034 (4)	0.016 (3)
O62	0.062 (4)	0.158 (6)	0.062 (3)	0.001 (4)	0.032 (3)	−0.026 (4)
O63	0.062 (3)	0.072 (3)	0.075 (3)	−0.001 (3)	0.034 (3)	0.009 (3)
N61	0.042 (4)	0.065 (4)	0.045 (4)	0.007 (3)	0.012 (3)	0.012 (3)
O64	0.081 (4)	0.079 (4)	0.128 (6)	0.012 (4)	0.025 (4)	−0.004 (4)
O65	0.065 (4)	0.254 (10)	0.112 (6)	−0.004 (5)	0.053 (4)	−0.001 (6)
O66	0.170 (8)	0.114 (6)	0.084 (5)	−0.013 (5)	0.062 (5)	0.005 (5)
N62	0.069 (5)	0.064 (4)	0.049 (4)	0.001 (4)	0.023 (4)	−0.008 (4)

### Geometric parameters (Å, º)

**Table:**

O4—C4	1.332 (8)	O36—H36	0.8200
O4—C21	1.411 (8)	O36—C36	1.344 (7)
O4*—H4*	0.8200	O37—C37	1.438 (7)
O4*—C4*	1.417 (7)	O37—C52	1.400 (7)
O5—C5	1.243 (7)	O39—H39	0.8200
O5*—C1*	1.405 (7)	O39—C39	1.429 (7)
O5*—C5*	1.435 (7)	O41—H41	0.8200
O6—H6	0.8200	O41—C41	1.351 (7)
O6—C6	1.351 (7)	O42—C42	1.243 (7)
O7—C1*	1.427 (7)	O43—C43	1.190 (7)
O7—C7	1.441 (6)	O44—H44	0.8200
O9—H9	0.8200	O44—C44	1.409 (7)
O9—C9	1.439 (7)	O55—H55	0.8200
O11—H11	0.8200	O55—C55	1.440 (7)
O11—C11	1.347 (7)	O56—C52	1.420 (7)
O12—C12	1.247 (7)	O56—C56	1.434 (7)
O13—C13	1.198 (7)	N54—H54A	0.8900
O14—H14	0.8200	N54—H54B	0.8900
O14—C14	1.394 (7)	N54—H54C	0.8900
N3*—H3*A	0.8900	N54—C54	1.506 (8)
N3*—H3*B	0.8900	C31—H31	0.9300
N3*—H3*C	0.8900	C31—C32	1.382 (9)
N3*—C3*	1.495 (7)	C31—C45	1.391 (8)
C1—H1	0.9300	C32—H32	0.9300
C1—C2	1.373 (9)	C32—C33	1.372 (10)
C1—C15	1.403 (8)	C33—H33	0.9300
C1*—H1*	0.9800	C33—C34	1.370 (10)
C1*—C2*	1.513 (9)	C34—C46	1.408 (8)
C2—H2	0.9300	C35—C46	1.474 (8)
C2—C3	1.346 (10)	C35—C48	1.461 (8)
C2*—H2*A	0.9700	C36—C48	1.373 (8)
C2*—H2*B	0.9700	C36—C50	1.427 (8)
C2*—C3*	1.494 (8)	C37—H37	0.9800
C3—H3	0.9300	C37—C38	1.499 (8)
C3—C4	1.395 (9)	C37—C50	1.496 (8)
C3*—H3*	0.9800	C38—H38A	0.9700
C3*—C4*	1.529 (8)	C38—H38B	0.9700
C4—C16	1.420 (8)	C38—C39	1.524 (8)
C4*—H4*A	0.9800	C39—C40	1.515 (8)
C4*—C5*	1.512 (8)	C39—C43	1.533 (8)
C5—C16	1.476 (8)	C40—H40A	0.9700
C5—C18	1.452 (8)	C40—H40B	0.9700
C5*—H5*	0.9800	C40—C49	1.512 (8)
C5*—C6*	1.501 (8)	C41—C47	1.393 (8)
C6—C18	1.390 (8)	C41—C49	1.403 (8)
C6—C20	1.416 (8)	C42—C45	1.460 (8)
C6*—H6*A	0.9600	C42—C47	1.445 (8)
C6*—H6*B	0.9600	C43—C44	1.482 (8)
C6*—H6*C	0.9600	C44—H44A	0.9700
C7—H7	0.9800	C44—H44B	0.9700
C7—C8	1.521 (7)	C45—C46	1.405 (9)
C7—C20	1.508 (7)	C47—C48	1.412 (8)
C8—H8A	0.9700	C49—C50	1.361 (8)
C8—H8B	0.9700	C51—H51A	0.9600
C8—C9	1.519 (8)	C51—H51B	0.9600
C9—C10	1.510 (8)	C51—H51C	0.9600
C9—C13	1.512 (8)	C52—H52	0.9800
C10—H10A	0.9700	C52—C53	1.499 (9)
C10—H10B	0.9700	C53—H53A	0.9700
C10—C19	1.485 (8)	C53—H53B	0.9700
C11—C17	1.375 (8)	C53—C54	1.499 (9)
C11—C19	1.412 (8)	C54—H54	0.9800
C12—C15	1.464 (8)	C54—C55	1.518 (8)
C12—C17	1.448 (8)	C55—H55A	0.9800
C13—C14	1.472 (9)	C55—C56	1.493 (8)
C14—H14A	0.9700	C56—H56	0.9800
C14—H14B	0.9700	C56—C57	1.501 (8)
C15—C16	1.404 (8)	C57—H57A	0.9600
C17—C18	1.426 (8)	C57—H57B	0.9600
C19—C20	1.377 (8)	C57—H57C	0.9600
C21—H21A	0.9600	O61—N61	1.221 (7)
C21—H21B	0.9600	O62—N61	1.228 (7)
C21—H21C	0.9600	O63—N61	1.237 (7)
O34—C34	1.361 (8)	O64—N62	1.212 (8)
O34—C51	1.414 (9)	O65—N62	1.200 (9)
O35—C35	1.231 (7)	O66—N62	1.223 (8)
			
C4—O4—C21	119.3 (5)	C39—O39—H39	109.5
C4*—O4*—H4*	109.5	C41—O41—H41	109.5
C1*—O5*—C5*	114.8 (4)	C44—O44—H44	109.5
C6—O6—H6	109.5	C55—O55—H55	109.5
C1*—O7—C7	112.8 (4)	C52—O56—C56	112.0 (4)
C9—O9—H9	109.5	H54A—N54—H54B	109.5
C11—O11—H11	109.5	H54A—N54—H54C	109.5
C14—O14—H14	109.5	H54B—N54—H54C	109.5
H3*A—N3*—H3*B	109.5	C54—N54—H54A	109.5
H3*A—N3*—H3*C	109.5	C54—N54—H54B	109.5
H3*B—N3*—H3*C	109.5	C54—N54—H54C	109.5
C3*—N3*—H3*A	109.5	C32—C31—H31	120.4
C3*—N3*—H3*B	109.5	C32—C31—C45	119.3 (7)
C3*—N3*—H3*C	109.5	C45—C31—H31	120.4
C2—C1—H1	121.4	C31—C32—H32	119.7
C2—C1—C15	117.2 (7)	C33—C32—C31	120.6 (6)
C15—C1—H1	121.4	C33—C32—H32	119.7
O5*—C1*—O7	112.7 (4)	C32—C33—H33	119.7
O5*—C1*—H1*	108.3	C34—C33—C32	120.5 (7)
O5*—C1*—C2*	111.2 (5)	C34—C33—H33	119.7
O7—C1*—H1*	108.3	O34—C34—C33	123.0 (6)
O7—C1*—C2*	108.0 (5)	O34—C34—C46	116.0 (6)
C2*—C1*—H1*	108.3	C33—C34—C46	121.0 (7)
C1—C2—H2	118.4	O35—C35—C46	122.1 (6)
C3—C2—C1	123.2 (7)	O35—C35—C48	119.1 (6)
C3—C2—H2	118.4	C48—C35—C46	118.7 (5)
C1*—C2*—H2*A	109.2	O36—C36—C48	122.1 (5)
C1*—C2*—H2*B	109.2	O36—C36—C50	116.8 (5)
H2*A—C2*—H2*B	107.9	C48—C36—C50	121.1 (5)
C3*—C2*—C1*	112.2 (5)	O37—C37—H37	108.0
C3*—C2*—H2*A	109.2	O37—C37—C38	112.6 (4)
C3*—C2*—H2*B	109.2	O37—C37—C50	106.9 (4)
C2—C3—H3	119.6	C38—C37—H37	108.0
C2—C3—C4	120.9 (6)	C50—C37—H37	108.0
C4—C3—H3	119.6	C50—C37—C38	113.2 (5)
N3*—C3*—H3*	108.3	C37—C38—H38A	108.8
N3*—C3*—C4*	110.0 (5)	C37—C38—H38B	108.8
C2*—C3*—N3*	111.5 (5)	C37—C38—C39	114.0 (5)
C2*—C3*—H3*	108.3	H38A—C38—H38B	107.7
C2*—C3*—C4*	110.3 (5)	C39—C38—H38A	108.8
C4*—C3*—H3*	108.3	C39—C38—H38B	108.8
O4—C4—C3	123.2 (6)	O39—C39—C38	113.3 (5)
O4—C4—C16	118.2 (6)	O39—C39—C40	110.9 (5)
C3—C4—C16	118.6 (6)	O39—C39—C43	104.0 (4)
O4*—C4*—C3*	110.8 (5)	C38—C39—C43	108.7 (5)
O4*—C4*—H4*A	110.1	C40—C39—C38	110.2 (5)
O4*—C4*—C5*	108.7 (5)	C40—C39—C43	109.5 (5)
C3*—C4*—H4*A	110.1	C39—C40—H40A	108.7
C5*—C4*—C3*	107.1 (5)	C39—C40—H40B	108.7
C5*—C4*—H4*A	110.1	H40A—C40—H40B	107.6
O5—C5—C16	122.0 (5)	C49—C40—C39	114.3 (5)
O5—C5—C18	119.1 (5)	C49—C40—H40A	108.7
C18—C5—C16	118.8 (5)	C49—C40—H40B	108.7
O5*—C5*—C4*	110.6 (5)	O41—C41—C47	121.9 (5)
O5*—C5*—H5*	108.4	O41—C41—C49	117.4 (5)
O5*—C5*—C6*	107.1 (5)	C47—C41—C49	120.7 (5)
C4*—C5*—H5*	108.4	O42—C42—C45	119.9 (5)
C6*—C5*—C4*	113.8 (5)	O42—C42—C47	121.2 (6)
C6*—C5*—H5*	108.4	C47—C42—C45	118.8 (5)
O6—C6—C18	122.1 (5)	O43—C43—C39	121.3 (5)
O6—C6—C20	116.4 (5)	O43—C43—C44	121.2 (6)
C18—C6—C20	121.5 (5)	C44—C43—C39	117.5 (5)
C5*—C6*—H6*A	109.5	O44—C44—C43	108.9 (5)
C5*—C6*—H6*B	109.5	O44—C44—H44A	109.9
C5*—C6*—H6*C	109.5	O44—C44—H44B	109.9
H6*A—C6*—H6*B	109.5	C43—C44—H44A	109.9
H6*A—C6*—H6*C	109.5	C43—C44—H44B	109.9
H6*B—C6*—H6*C	109.5	H44A—C44—H44B	108.3
O7—C7—H7	108.7	C31—C45—C42	117.5 (6)
O7—C7—C8	111.6 (4)	C31—C45—C46	121.1 (6)
O7—C7—C20	107.1 (4)	C46—C45—C42	121.5 (5)
C8—C7—H7	108.7	C34—C46—C35	123.2 (6)
C20—C7—H7	108.7	C45—C46—C34	117.4 (6)
C20—C7—C8	111.9 (5)	C45—C46—C35	119.4 (6)
C7—C8—H8A	108.9	C41—C47—C42	120.1 (5)
C7—C8—H8B	108.9	C41—C47—C48	119.3 (5)
H8A—C8—H8B	107.7	C48—C47—C42	120.6 (5)
C9—C8—C7	113.5 (5)	C36—C48—C35	120.0 (5)
C9—C8—H8A	108.9	C36—C48—C47	119.4 (5)
C9—C8—H8B	108.9	C47—C48—C35	120.7 (5)
O9—C9—C8	111.2 (5)	C41—C49—C40	117.9 (5)
O9—C9—C10	110.3 (5)	C50—C49—C40	121.8 (5)
O9—C9—C13	104.3 (4)	C50—C49—C41	120.3 (5)
C10—C9—C8	109.0 (4)	C36—C50—C37	118.2 (5)
C10—C9—C13	111.7 (5)	C49—C50—C36	119.2 (5)
C13—C9—C8	110.2 (5)	C49—C50—C37	122.6 (5)
C9—C10—H10A	108.8	O34—C51—H51A	109.5
C9—C10—H10B	108.8	O34—C51—H51B	109.5
H10A—C10—H10B	107.7	O34—C51—H51C	109.5
C19—C10—C9	114.0 (5)	H51A—C51—H51B	109.5
C19—C10—H10A	108.8	H51A—C51—H51C	109.5
C19—C10—H10B	108.8	H51B—C51—H51C	109.5
O11—C11—C17	121.9 (5)	O37—C52—O56	111.6 (5)
O11—C11—C19	116.3 (5)	O37—C52—H52	107.8
C17—C11—C19	121.8 (5)	O37—C52—C53	109.0 (5)
O12—C12—C15	119.7 (5)	O56—C52—H52	107.8
O12—C12—C17	120.1 (5)	O56—C52—C53	112.7 (5)
C17—C12—C15	120.2 (5)	C53—C52—H52	107.8
O13—C13—C9	121.1 (5)	C52—C53—H53A	109.1
O13—C13—C14	121.0 (6)	C52—C53—H53B	109.1
C14—C13—C9	117.9 (5)	H53A—C53—H53B	107.8
O14—C14—C13	115.3 (6)	C54—C53—C52	112.6 (5)
O14—C14—H14A	108.5	C54—C53—H53A	109.1
O14—C14—H14B	108.5	C54—C53—H53B	109.1
C13—C14—H14A	108.5	N54—C54—H54	108.6
C13—C14—H14B	108.5	N54—C54—C55	109.6 (5)
H14A—C14—H14B	107.5	C53—C54—N54	110.3 (5)
C1—C15—C12	117.6 (6)	C53—C54—H54	108.6
C1—C15—C16	121.5 (6)	C53—C54—C55	111.1 (5)
C16—C15—C12	120.8 (5)	C55—C54—H54	108.6
C4—C16—C5	122.1 (6)	O55—C55—C54	108.8 (5)
C15—C16—C4	118.4 (6)	O55—C55—H55A	109.7
C15—C16—C5	119.5 (5)	O55—C55—C56	111.6 (5)
C11—C17—C12	120.8 (5)	C54—C55—H55A	109.7
C11—C17—C18	120.6 (5)	C56—C55—C54	107.4 (5)
C18—C17—C12	118.5 (5)	C56—C55—H55A	109.7
C6—C18—C5	121.0 (5)	O56—C56—C55	108.3 (5)
C6—C18—C17	117.2 (5)	O56—C56—H56	108.9
C17—C18—C5	121.8 (5)	O56—C56—C57	109.0 (5)
C11—C19—C10	118.6 (5)	C55—C56—H56	108.9
C20—C19—C10	123.4 (5)	C55—C56—C57	112.9 (5)
C20—C19—C11	117.9 (5)	C57—C56—H56	108.9
C6—C20—C7	118.2 (5)	C56—C57—H57A	109.5
C19—C20—C6	120.7 (5)	C56—C57—H57B	109.5
C19—C20—C7	121.1 (5)	C56—C57—H57C	109.5
O4—C21—H21A	109.5	H57A—C57—H57B	109.5
O4—C21—H21B	109.5	H57A—C57—H57C	109.5
O4—C21—H21C	109.5	H57B—C57—H57C	109.5
H21A—C21—H21B	109.5	O61—N61—O62	122.5 (7)
H21A—C21—H21C	109.5	O61—N61—O63	119.6 (7)
H21B—C21—H21C	109.5	O62—N61—O63	117.8 (7)
C34—O34—C51	118.6 (6)	O64—N62—O66	117.4 (8)
C36—O36—H36	109.5	O65—N62—O64	120.8 (9)
C52—O37—C37	114.0 (5)	O65—N62—O66	121.8 (9)

### Hydrogen-bond geometry (Å, º)

Cg2 is the centroid of the C1–C4/C15/C16 ring.

**Table:**

*D*—H···*A*	*D*—H	H···*A*	*D*···*A*	*D*—H···*A*
O6—H6···O5	0.82	1.81	2.526 (6)	146
O11—H11···O12	0.82	1.80	2.526 (6)	146
O14—H14···O65^i^	0.82	2.07	2.779 (9)	145
N3*—H3**A*···O13^ii^	0.89	2.00	2.874 (7)	167
N3*—H3**C*···O63	0.89	1.99	2.865 (8)	168
O41—H41···O42	0.82	1.82	2.537 (6)	146
O44—H44···O14^iii^	0.82	2.08	2.888 (6)	168
O55—H55···O4*	0.82	1.93	2.724 (6)	163
N54—H54*A*···O43^iv^	0.89	2.18	2.890 (7)	136
N54—H54*A*···O44^iv^	0.89	2.05	2.843 (7)	147
N54—H54*B*···O62	0.89	1.99	2.836 (8)	159
N54—H54*C*···O64	0.89	2.05	2.876 (9)	155
C38—H38*B*···*Cg*2^v^	0.97	2.64	3.556 (7)	157

Symmetry codes: (i) *x*−1, *y*, *z*+1; (ii) *x*, *y*, *z*−1; (iii) *x*+2, *y*, *z*; (iv) *x*−1, *y*, *z*−1; (v) −*x*+1, *y*+1/2, −*z*+1.
